# Advances in Additive Manufacturing of Polymer-Fused Deposition Modeling on Textiles: From 3D Printing to Innovative 4D Printing—A Review

**DOI:** 10.3390/polym16050700

**Published:** 2024-03-04

**Authors:** Edgar Adrian Franco Urquiza

**Affiliations:** Advanced Manufacturing Department, Center for Engineering and Industrial Development, CIDESI-Airport, Carretera Estatal 200, km 23, Queretaro 76270, Mexico; edgar.franco@cidesi.edu.mx

**Keywords:** 3D printing polymers, additive manufacturing, 4D printing, polymer textiles, intelligent textiles

## Abstract

Technological advances and the development of new and advanced materials allow the transition from three-dimensional (3D) printing to the innovation of four-dimensional (4D) printing. 3D printing is the process of precisely creating objects with complex shapes by depositing superimposed layers of material. Current 3D printing technology allows two or more filaments of different polymeric materials to be placed, which, together with the development of intelligent materials that change shape over time or under the action of an external stimulus, allow us to innovate and move toward an emerging area of research, innovative 4D printing technology. 4D printing makes it possible to manufacture actuators and sensors for various technological applications. Its most significant development is currently in the manufacture of intelligent textiles. The potential of 4D printing lies in modular manufacturing, where fabric-printed material interaction enables the creation of bio-inspired and biomimetic devices. The central part of this review summarizes the effect of the primary external stimuli on 4D textile materials, followed by the leading applications. Shape memory polymers attract current and potential opportunities in the textile industry to develop smart clothing for protection against extreme environments, auxiliary prostheses, smart splints or orthoses to assist the muscles in their medical recovery, and comfort devices. In the future, intelligent textiles will perform much more demanding roles, thus envisioning the application fields of 4D printing in the next decade.

## 1. Introduction

3D printing technology, or additive manufacturing, has quickly become a design procedure used to manufacture parts or components that often contain complex geometries. Thus, the interest in 3D printing arises from the need for rapid prototyping and the creation of adjustable parts. Rapid prototyping allows iterations of different proposed concepts so that computer-generated designs can be manufactured relatively quickly and at low cost for further evaluation, usually on a test bench or pilot plant. In this way, creators develop innovations that could not be achieved with conventional machined parts or components [1,2,3].

The conceptual part is based on computer-aided design (CAD) to reduce the iterative development cycle. Iterative prototypes developed can be quickly replaced or modified as needed based on parts or user requirements. Therefore, 3D printing provides a large amount of customization in the structure of components and is even used to print components that cannot be machined with typical manufacturing methods in a relatively short time. For this reason, 3D printing technology has grown exponentially in recent years, becoming one of the conventional production methods for manufacturing individual components [1,4]. 3D printers create parts from three-dimensional models, which are mathematical representations of any three-dimensional surface created with computer-aided design (CAD) software or developed from 3D scan data. Once the part is released for manufacturing, the designs are exported as an STL or OBJ file, readable by print preparation software. Often unique to the type of 3D printing and even the brand of 3D printer, this software specifies print settings and divides the digital model into layers representing cross and horizontal sections of the part. Adjustable print settings include orientation, support structures (if necessary), layer height and material, and generating routing instructions for the 3D printer to build the model layer by layer. After configuration, the software sends the instructions to the printer through a wireless or wired connection. All 3D printing processes start with a CAD model sent to software to prepare the design. Depending on the technology, the 3D printer can produce the part layer by layer by solidifying the resin or sintering the powder. The parts are then removed from the printer and post-processed for the specific application.

3D printing technology is inherently digital, encompassing various 3D printing processes that use entirely different machines and materials (polymers, metals, and ceramics) but with typical critical elements, like being able to print anything from pencil holders to propulsion engine sections. There is a diversity of raw materials used for 3D printing technologies. Depending on the technology, the development of materials can be diverse and exponential. Increasing attention is paid to developing high-performance polymers (smart polymers and polymer compounds with structural properties) for infrastructure, energy, communications, and technology use. Thus, the additive manufacturing of polymers has become a fundamental technological process towards the fourth industrial revolution in various fields of application [5,6,7,8,9,10,11,12].

**Figure 1:**
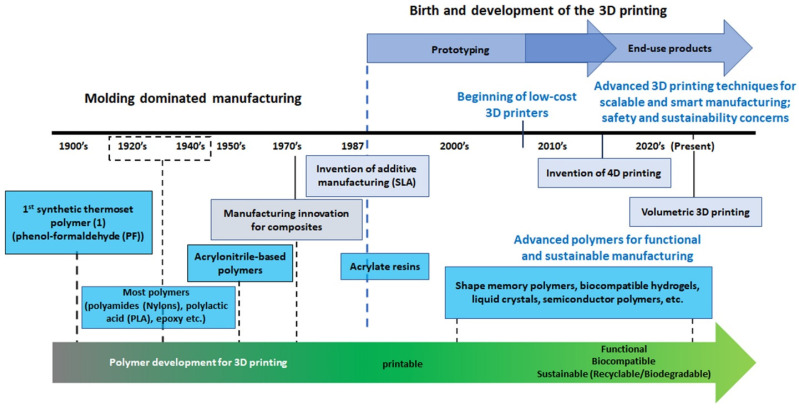
Timeline from rapid prototyping to scalable and customizable production [13]. Reproduced from [13], with permission from Elsevier and Copyright Clearance Center, 2023.

Soyeon et al. presented a timeline of 3D printing alongside polymer development, as shown in Figure 1.

Today, it is possible to find research papers documenting the properties of experimental materials used in 3D printing. Most of these materials are developed in universities and research centers, with excellent results. However, the availability of commercial materials is wide, depending on the 3D printing technology used. The polymers used in 3D printing are thermoplastic and thermosetting resins, each with unique qualities that make it more suitable for the available technology. Thermoplastics (semi-crystalline and amorphous) can go through numerous melting and solidification cycles to be recycled, because the solidification process is reversible and no chemical bond occurs, although each process entails a decrease in properties due to degradation. Thermosetting plastics crosslink during a curing process induced by heat, light, or suitable radiation. Thermosetting plastics have no recyclability due to their crosslinked structure, which leads to their excellent engineering properties and only breaks down with increasing temperature.

This article reports the most used polymers in the various 3D printing technologies today. This review article aims to summarize the progress of 3D printing of polymers towards its application in 4D printing in textiles, highlighting some of the most outstanding challenges and opportunities so far, showing the foreseeable evolution for the coming years and the field of opportunities that arise for industry, academia, and entrepreneurs.

## 2. 3D Printing Materials

Today, a wide variety of materials are used for Fused Deposition Modeling (FDM) printing. Acrylonitrile butadiene styrene (ABS) and polylactic acid (PLA) are the most common materials.

ABS is the most widely used 3D printing thermoplastic due to its versatility and mechanical properties. ABS combines the resilience of butadiene and the stiffness of styrene, where the ratio of the two allows the properties of ABS to be modified to improve its toughness and impact resistance. The versatility of ABS allows it to be used as a filament, powder, or liquid [14,15].

Serena Rifuggiato et al. highlighted the importance of non-destructive inspection in the quality control of 3D-printed parts by relating defects induced by the 3D printing process and the mechanical performance of printed parts. The authors performed fill tests to simulate mechanical resistance. Using computed tomography, the authors visualized the various imperfections of the 3D printing process prior to its mechanical testing. In this way, the results allowed us to conclude that the presence of defects (internal voids) favored a reduction in mechanical performance, even though the failure did not coincide with the areas of the most prominent defects. These results allow us to visualize the opportunities to evaluate 3D-printed parts in terms of fracture mechanics [16,17].

PLA is a biodegradable plastic synthesized from renewable raw materials such as corn starch [18] and is the most popular 3D printing filament, although it tends to shrink due to its structure [19]. S. Hamat et al. studied the effects of 3D printing parameters with PLA on the printed parts’ mechanical performance (tensile strength). The results revealed that the optimal printing temperature and speed, which achieve the highest mechanical resistance, are around 175 °C and 4 rpm, respectively.

The 3D printing process using polymer filaments opens a window of research opportunities in the search for disruptive solutions. For example, it is possible to incorporate drugs before 3D printing parts for the health sector, such as medical devices or splints printed to measure patients [20]. For this reason, there is considerable interest in using the Design of Experiments (DOE) to optimize 3D printing with PLA. Luca Fontana et al. applied the DOE on parts printed with PLA, finding that the thickness of the layers is more significant than the fill percentage for mechanical resistance. However, various investigations detail the effects of humidity, temperature, and pressure on the mechanical properties of PLA [18,19]. PLA is affected by multiple environmental factors and processing and transformation conditions. This way, R. Jain and N. Gupta [21] developed a DOE to examine the optimization of PLA 3D printing, using design factors such as unit cell and lattice size, layer height, filament print speed and temperature, density, and pattern of the fill, types of support, temperature of construction and orientation, and height of the layer. The objective was to reveal influential parameters to increase the mechanical resistance of parts printed in 3D with PLA.

In general, recent scientific publications detail that the mechanical properties of 3D-printed parts through Fused Filament Fabrication (FFF) or FMD are strongly influenced by the thickness of the layer, the degree of filling, and the anisotropy of the pattern. Lokesh N. et al. [22] studied the effect of layer thickness, build orientation, and screen angle on the mechanical properties of 3D-printed PLA. The authors used the Taguchi approach with the L9 orthogonal matrix and analyzed variance to test for potential significant features, finding that the layer thickness has more influence than the build orientation or the raster angle.

The diversity of using polymeric filaments for 3D printing under FFF or FDM techniques is vast. It has been seen that engineering polymers such as ABS or biodegradable plastics such as PLA are used. However, making 3D-printed parts, using other conventional and recyclable polymers, is also possible. Polyethylene terephthalate (PET) is the most common synthetic polyester (PLA is also a thermoplastic polyester). PET filament is used in 3D printing parts with excellent chemical resistance and considerable mechanical properties [4,23]. Additionally, PET is an FDA-approved material for food packaging, making it ideal for any part intended to come into contact with food and is recyclable, giving it an open window to use in prototypes. PET can be synthesized with ethylene glycol (PETG) to increase its ductility [24,25,26].

PET is used in various markets, although it is mainly used to manufacture textiles and bottles, with the latter being recognized as disposable products. Much of the PET is recycled to transform it into new products, so it would not be strange to use recycled PET in 3D printing, even more so considering that PET is easy to extrude. Despite this, PET in 3D printing is not widespread today, mainly due to its semi-crystalline nature. Some authors [4,27,28,29] highlight that semi-crystalline polymers often hinder 3D printing. For this reason, recent investigations detail the use of modified polyethylene terephthalate glycol (PETG) in 3D printing, since substituting ethylene glycol for cyclohexanedimethanol in the PET skeleton makes it more flexible and less brittle when printed.

Alaeddine Oussai et al. [30] manufactured pure PET and recycled PET-extruded filaments to compare their mechanical properties and found no substantial differences. The authors printed test tubes with the same 3D printing characteristics and found that recycled PET leads to better mechanical properties without influencing print quality compared to pure PET, which exhibited better tensile behavior. Both materials kept their stiffness unchanged. Based on these results, the authors highlighted the importance of controlling the manufacturing process of PET filaments, since extrusion conditions can induce the degradation of the material [31].

Polycarbonate (PC) is a highly resistant material oriented towards engineering applications due to its transparency and excellent mechanical and thermal resistance. PC is one of the materials beginning to be used in 3D printing due to its mechanical properties and dimensional stability. Similarly, 3D printing parameters such as screen angle, air gap, and screen width have been studied to better understand and optimize the 3D printing process [32,33,34]. Like ABS, which can be combined with other materials, PC is combined with other thermoplastic polymers or inorganic particles [35] to take advantage of the intrinsic mechanical properties between materials and improve the performance of printed parts. Most of the research is mainly based on combining PC with ABS [36,37,38,39] and PET [40].

Nylon is a polymer from the synthetic polyamide family, classified as a high-engineering plastic due to its high durability, resistance, and flexibility. Because it offers excellent mechanical properties and good chemical impact and heat resistance, nylon 3D printing is ideal for functional parts and prototypes, which is why it is used in various printing technologies, such as fused deposition modeling (FDM) [41]. Polyamides also present a subclassification according to the number of carbons in their structure, and therefore with different properties between them, known as Polyamide 6, Polyamide 66, Polyamide 11, and Polyamide 12. In this way, the use of nylon in 3D printing will depend on the type of polyamide being used. Some polyamides, such as nylon 618, provide better wear performance due to differences in crystallinity and the uniqueness of the FDM process [41]. Other authors established that higher crystallinity could increase elasticity when polyamides are heated beyond the glass transition temperature [42]. Despite their versatility, polyamides are often susceptible to absorbing atmospheric moisture, which can affect their performance [43]. In addition, nylon is often reinforced with fibers and particles of other materials to improve its thermal and mechanical properties, such as stiffness or abrasion resistance. Tavcar et al. [44] investigated the service life of various materials and reinforced materials, including nylon, nylon 66, Polyoxymethylene (POM), and Polyphenylene sulfide (PPS), and the results showed that reinforced materials could survive more cycles if lubrication were applied to them. Another available material is styrene-acrylonitrile acrylate (ASA), a material with properties similar to ABS but with more excellent resistance to UV rays, tolerance to temperature changes, and chemical resistance [45,46,47].

On the other hand, composite materials with different reinforcements have many applications [48]. Abdul Zubar Hameed et al. [49] evaluated and confirmed that an adequate configuration of the process and the added fibers and fillers can improve the properties of the composite. Composite materials in additive manufacturing can use thermoplastic polymers with recycling capacity and thermosetting polymers that cannot be recycled but have structural properties [50].

The resins used for photocuring 3D printing are known as photosensitive resins and generally use SLA technology. Parts printed with SLA 3D printing are generally isotropic. Light-curing 3D printing is primarily used for manufacturing highly detailed prototypes that require tight tolerances and smooth surfaces, such as molds, patterns, and functional parts. Photoresists can be standard, transparent, semi-transparent, casting resins, hard and resistant resins, high-temperature resistant, biocompatible, abrasion-resistant, or flexible resins. Each of these materials has properties and characteristic features that allow for their selection, considering the requirements of the part to be printed [29,51]. The above-mentioned factors should also consider the printing technology and the wavelength of the lamp, mainly.

## 3. 3D Printing Technologies

3D printing technology has gained great popularity recently due to its simple and diversified technology. Its use has spread to multiple fields of knowledge, covering education, prototyping, construction, medicine, and the aerospace industry, among many others. 3D printing enables the development of lightweight parts for fuel and energy efficiency. In addition, the technology also allows parts to be created from materials customized for specific properties, such as water repellency, increased strength, and heat resistance.

ISO/ASTM 52900:2021 [52] establishes and defines the terms used in additive manufacturing technology and classifies general additive manufacturing technologies into seven types, considering the type of material used and the final application of the printed parts:

1. Extrusion of materials
2. Tub polymerization
3. Powder bed fusion
4. Material jet
5. Blast blast
6. Directed Energy Deposition (Metal Alloys)
7. Rolling sheet

**Figure 2:**
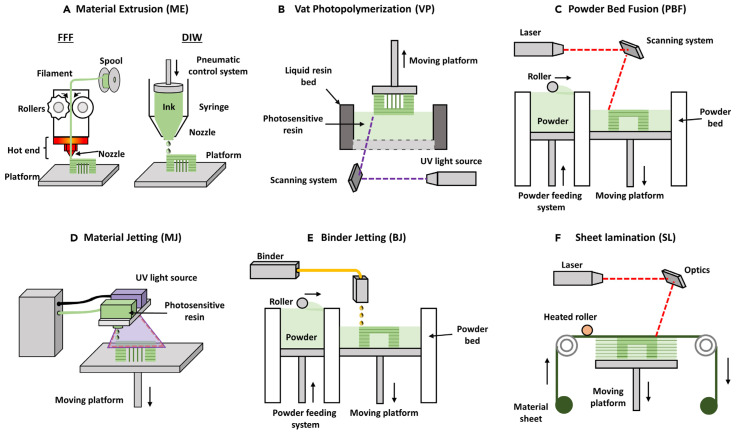
Schematic representations of 3D printing techniques [13]. Reproduced from [13], with permission from Elsevier and Copyright Clearance Center, 2023.

However, these seven categories of 3D printing are constantly evolving to encompass the growing variety of hybrid technologies and applications projected for the next decade [27,28]. Figure 2 presents an overview of the different additive manufacturing technologies according to the configuration of the materials [13].

### 3.1. Extrusion Material

Extrusion technology involves melting the polymer and passing it through a nozzle. In this way, the extruded material turns out to be a plastic filament deposited on a platform along a path determined by the build preparation software. The filament then cools and solidifies to form a solid object. This is the most common form of 3D printing, so it is affordable due to its cost and the wide range of materials available. This technology’s weakness lies in the material’s reduced properties and its low precision in terms of the dimensional dimensions of the CAD design. As previously mentioned, the FDM or FFF additive manufacturing processes deposit the molten plastic layer by layer until the part is built and are currently the most widely used due to their low cost and ease of access.

### 3.2. Vat Polymerization

Vat polymerization, or 3D printing of resin, uses a light source to selectively cure liquid photopolymer resin in a vat. In other words, the light is directed precisely at a specific point or area of the liquid plastic to harden it. Once the first layer is cured, the build platform is moved up or down (depending on the printer), and the next layer is cured, joining the previous one. This process is repeated layer by layer until the 3D part is formed. Once the 3D printing process is complete, the object is cleaned to remove any traces of liquid resin and is post-cured, usually in an ultraviolet chamber, to improve the mechanical properties of the part as a result of complete curing.

### 3.3. Powder Bed Fusion

Powder Bed Fusion (PBF) is a 3D printing process in which a thermal energy source selectively melts powder particles (plastic, metal, or ceramic) within a build area to create a solid object layer by layer. Powder bed fusion 3D printers spread a thin layer of powdered material onto the print bed, typically with a blade, roller, or wiper. Energy, usually from a laser, fuses specific points in the layer of dust, and then, another layer of dust is deposited and merges with the previous layer. The process is repeated until the entire object is manufactured. The final item is encased and supported in unfused powder. Although the process varies depending on whether the material is plastic or metal, PBF can create parts with high mechanical properties, including strength, wear resistance, and durability, for end-use applications in consumer products, machinery, and tools. Although 3D printers in this segment are becoming more affordable, they are considered professional or industrial technology.

### 3.4. Material Stream

In this 3D printing process, small droplets of material are deposited and then solidified or cured on a build plate. Pieces are built layer by layer using photopolymers that cure when exposed to light. This 3D printing process is flexible with the materials used, allowing different materials to be printed on the same part. Once a layer has been deposited and cured, the build platform is lowered to one layer thickness, and the process is repeated to build a 3D object.

### 3.5. Binder Blast

In this 3D printing process, a liquid bonding agent selectively bonds regions of a layer of powder. The type of technology has the qualities of powder bed fusion and material jetting. Similar to PBF, binder jetting uses a powdered material (metal, plastic, ceramic, wood, sugar), and, like media jetting, a liquid binder polymer is deposited from the ink on the PBF jets. The binder injection process is the same whether for metal, plastic, sand, or other powdered material.

### 3.6. Directed Energy Deposition

Directed Energy Deposition (DED) is a unique 3D printing process for metal alloys and encompasses a wide range of derived technologies, depending on the configuration of the material (wire or powder) and the type of energy.

### 3.7. Sheet Lamination

Foil lamination is technically a form of 3D printing, although it differs drastically from previous technologies. It works by stacking and laminating sheets of very thin material to produce a 3D object or stack that is mechanically or laser-cut to form the final shape. Polymers and metals are mainly used for this printing technique, although they can be used to produce composite materials since they can be interchanged during the printing process. Although the industry uses sheet lamination to produce cost-effective, non-functional prototypes at a relatively high rate, more scraps are generated after machining compared to other 3D printing technologies.

For the technologies shown above, the raw materials for 3D printing are filaments, liquid and colloid resins, powders, and solid sheets [53]. The selection of materials depends on the printing technology adopted and ranges from thermoplastics, thermosets, hydrogels, and conductive materials [54]. Additive manufacturing is primarily used for manufacturing custom parts, including prototypes and small series products. However, the 3D printing industry has experienced exponential growth in recent years. In this way, different additive manufacturing technologies are being adopted in robotics [55], biomedical [56], aeronautical [57], and self-powered device sectors [58]. 3D printing has gained considerable interest in academia and industry due to its potential in establishing smart manufacturing.

## 4. Additive Manufacturing in Textiles

The alternatives of 3D printing technologies are currently vast, and the knowledge generated over the last decade allows the field of application of additive manufacturing (AM) to expand to other sectors, such as the textile industry. It is essential to highlight that when discussing the textile industry, its application is mainly perceived in clothing or footwear. However, the extensive and varied additive manufacturing technology, nanotechnological advances, and the creation of new materials in laboratories allow the concept of textiles to be extended to biomedical, aerospace, sports, and infrastructure applications, among many others. This review work is limited to using FFF or FDM technology in manufacturing textiles.

Textile additive manufacturing has the potential to significantly reduce the amount of resources needed to produce the fabrics used in clothing or footwear. Manufacturing processes can be agile when transforming complex fabrics, but they also guarantee the optimization and sustainable consumption of raw materials, chemical products, and water. Mixed-material printing capabilities now exist that provide opportunities for advanced and innovative material design that were not possible with traditional manufacturing techniques. Thus, textiles are being addressed towards additive manufacturing in several forms and functions due to their potential to improve the complexity and functionality of yarns or filaments [59,60].

One of the most cost-effective ways to use 3D printing on textiles is to print patterns onto fabrics. However, it is necessary to consider the adhesion between the polymer filament and the substrate. It has previously been seen that one of the critical factors in the FFF or FDM techniques is the adhesion between the layers. Korger et al. [61] evaluated the adhesion and stability of 3D printing on synthetic fabrics, finding optimal adhesion when performing peel and abrasion resistance tests.

Other researchers evaluated adhesion by comparing the behavior of polymeric filaments such as ABS, nylon, and PLA [62]. Pei et al. [63] detailed that PLA has the best adhesion properties.

Other approaches involve creating bi-material 3D-printed objects in which tissue is trapped between the printed layers [64]. Some textile fabrics contain gaps or holes between the warp and weft threads in the weave pattern, so the top layer placed over such fabrics can snag the bottom layer through those gaps. Some researchers found that poor adherence arises with closed tissues [65].

Printing an entire item of clothing is much more time-consuming and expensive than typically creating the item and adding 3D-printed elements when necessary. Tomasz Kozior et al. [66] pretreated a cotton fabric to assess the adhesion forces between the pretreated fabric and a PLA 3D-printed pattern to elucidate whether hydrophobicity alters the resulting adhesion forces [67].

**Figure 3:**
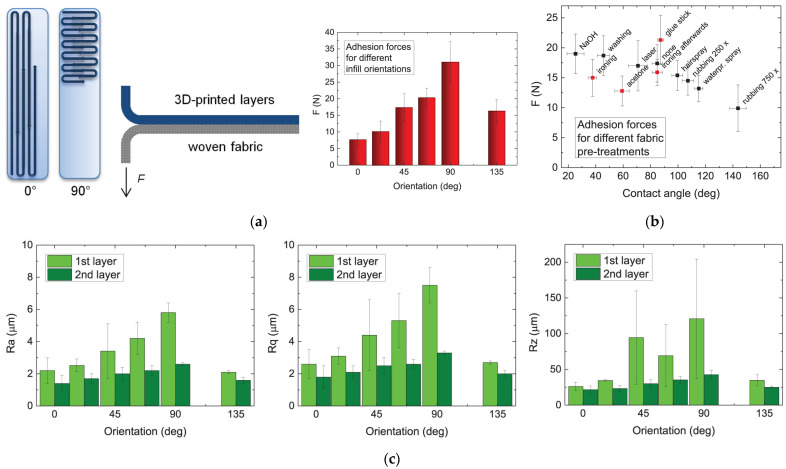
Fill orientations and adhesion forces in 3D printing [66]: (**a**) infill orientations, (**b**) adhesion test, and (**c**) roughness values Ra, Rq, and Rz measured for different infill orientations of the 3D printed samples. Adapted from [66] under the terms of SAGE and Creative Commons Attribution 4.0 License, 2023.

Efforts were directed towards modifying the fill angles to examine the surface roughness of the first and second printed layers, which few researchers have explored thus far with textile fabrics [66,68]. Figure 3a shows how fill orientations are derived and their effect on adhesion forces when PLA is additively manufactured on cotton fabric. The results indicated no significant changes in the mechanical properties of this kind of composite, although the surface roughness depends on the fill angle. Additionally, it was found that the more hydrophilic the fabric, the greater the adhesion force with the PLA filament and vice versa (Figure 3b). Regarding the contact angle, the authors noted that the adhesion forces seem to saturate below a contact angle of approximately 60°–80°. In contrast, for increasing contact angles, the adhesion forces decrease steadily [66], demonstrating the importance of considering the effects of fabric pretreatment, as presented in Figure 3c.

Franco-Urquiza et al. [68] evaluated the feasibility of fabricating PLA composites on jute plant fiber-woven fabrics. The jute-woven fabric contains a particular opening in the weft, implying a deposition of the PLA filament on each layer, ensuring the encapsulation and three-dimensional interaction of the novel composite. The jute fabrics were modified to have higher molecular adhesion with PLA. Despite the different strategies used, the interaction between printed PLA and jute fabric was insufficient and led to a more detailed investigation of the effect of plant fiber fabrics as reinforcements of 3D-printed objects for use in industrial applications.

Instead of additive manufacturing on textiles, another approach is to print the textiles themselves. Although it is not economically viable at an industrial level, it is justified for experimental tests. Giselle Hsiang Loh et al. [69] developed polymer and textile tests using PLA filaments in direct extrusion synthetic mesh fabrics. The authors evaluated the proper combination of impression material, textile substrate, and printer settings to achieve excellent polymer–textile adhesion. Testing was performed under the tear setting to measure the peel strengths of different textile composites and bonded polymers to assess material compatibility.

The additive manufacturing results in textiles allow us to glimpse that selecting adequate materials that allow adequate molecular interaction between the substrate and the polymeric filament provides relative success in adhesion. However, behavior and optimal performance require further understanding. There are still several questions regarding the interaction of both materials, such as the influence of micro- and mesoscale textile parameters [70]. Additionally, the 3D printing process must be evaluated in detail to understand the influence of FDM or FFF parameters, such as the temperature of the thermal base and the nozzle, flow rate, production speed, and the distance between the nozzle and the tissue, among others [60,71]. In order to ensure comparable data, standardized test procedures must be developed. Based on experimental data, models can be derived that lead to a better understanding of the influence of textile and process parameters on the adhesion and mechanical properties of 4D textile hybrid material.

## 5. From 3D to Innovative 4D Printing Polymer Textiles

**Figure 4:**
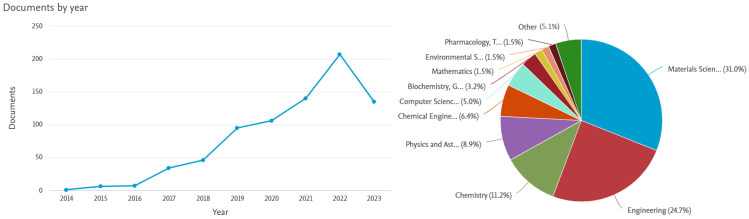
Analysis of search results of research articles in SCOPUS. Keywords: “4D printing” and “polymers”.

Additionally, three-dimensional printing technology has constantly improved and evolved substantially over the last three decades, giving rise to the beginnings of 4D printing. The first study on 4D printing polymers was published in 2014 [72], according to the search performed in SCOPUS, and it has been growing exponentially in recent years, as shown in Figure 4.

The term “4D printing” is attributed to Skylar, who demonstrated at a TED conference at MIT in 2012 [73] how transformations made to a 3D-printed object might change its shape over time, introducing the fourth dimension associated with 4D printing. 4D printing uses materials capable of changing shape and producing physical changes when exposed to ultraviolet and visible light [74], which means that 3D-printed objects can change shape over time and under external stimuli [75]. These stimuli include heat [76], electric fields [77], magnetic fields [78], light [79], water [80], and pH [81].

Due to the above, it is possible to define that the materials used in 4D printing are programmable and, therefore, intelligent since they respond to external or internal situations, changing the geometry of the 3D-printed structure and its properties and functionality. Innovative materials have been studied outside the field of additive manufacturing, but their results have allowed them to be combined to develop complex and multifunctional structures. Intelligent materials are also known as stimulus-responsive materials (SRMs) and can be designed to respond in a controllable and often reversible manner. SRMs are very dynamic in form and function [82]. Selecting the most suitable SRM is fundamental because it determines the type of stimulus necessary to trigger a response, which can be a change of property, shape, self-assembly, self-diagnosis, and self-repair [74,83]. For this reason, SRMs directly influence the mechanical properties and intelligence of the 4D-printed structure.

Shape memory polymers (SMPs), liquid crystal elastomers (LCEs), dielectrics, hydrogels, and their compounds are considered intelligent materials currently used for the 4D printing of multifunctional objects. It should be noted that intelligent materials can respond to one or more stimuli. Thus, innovative materials used in 4D printing can recover geometrically, conduct heat or electricity, and modify mechanical properties to function as sensors or actuators [84,85,86].

Based on the above, developing 4D printing polymer textiles from various approaches is possible. One of them is to print 3D textile structures using shape memory materials [87,88]. In this context, the shape change of textile structures due to an external stimulus has already shown great potential [8], as shape memory materials can be incorporated by weaving, sewing, or braiding.

Asterios Agkathidis and Guzden Varinlioglu [89] developed self-forming portable composite structures by 3D printing semi-elastic relief patterns from Thermoplastic Polyurethane 95 filaments. The authors generated two self-forming textile structures: the first is based on printed patterns related to their simulation of stress lines, and the second on modified geometric patterns in connection with curvature analysis.

**Figure 5:**
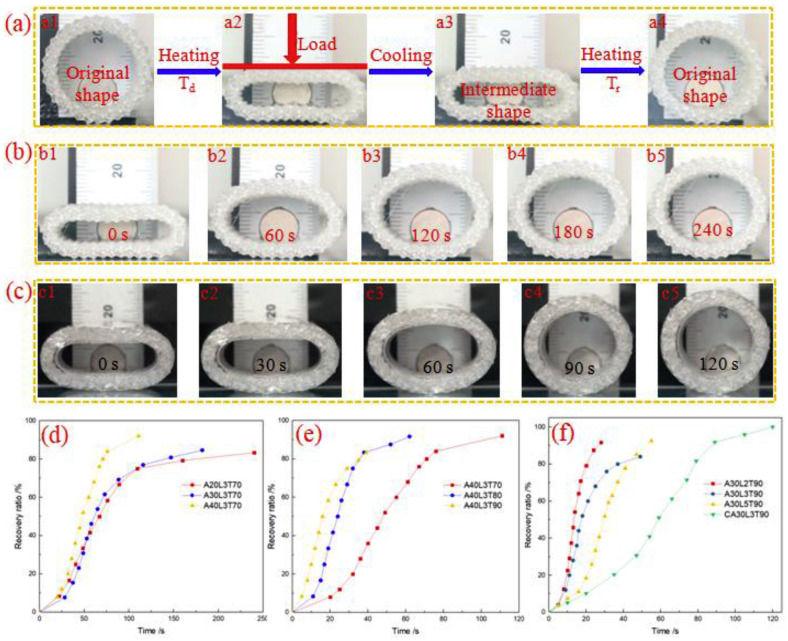
4D-printed circular braided tube shape memory cycle [90]: (**a**) Shape memory cycle of 4D printed circular braided tube, (**b**) shape recovery of the specimen A30L3T70, (**c**) shape recovery of specimen CA30L3T90, (**d**) shape recovery ratio vs. time curves of specimens L3T70 with three braiding angles of 20°, 30° and 40°, (**e**) shape recovery ratio vs. time curves of specimens A40L3 at three shape recovery temperatures, and (**f**) shape recovery ratio vs. time curves of specimens A30T90 with two braiding layers, three braiding layers and five braiding layers and the specimen CA30L3T90. Adapted from [90] under the terms of Elsevier and Copyright Clearance Center, 2023.

Zhang et al. [90] evaluated the shape memory and recovery force of 3D-printed preforms. The authors used silicone elastomer compounds. The work allows for evaluating the recovery force through an elastomer that changes its shape when applying a determined action, mainly influenced by the microstructural parameters (braiding angle and wall thickness) and the shape recovery. When the silicone elastomer is introduced, the radial compression failure face increases, and the same occurs with the shape recovery force, achieving this recovery in a much greater temperature range (Figure 5).

Another approach to creating textiles that change form and function is hybrid fabric-printed polymer composites, where the textile acts as a substrate that stores energy [63]. 3D printing can also apply conductive layers and fix electronic components on textile surfaces, which may be helpful in intelligent textiles.

**Figure 6:**
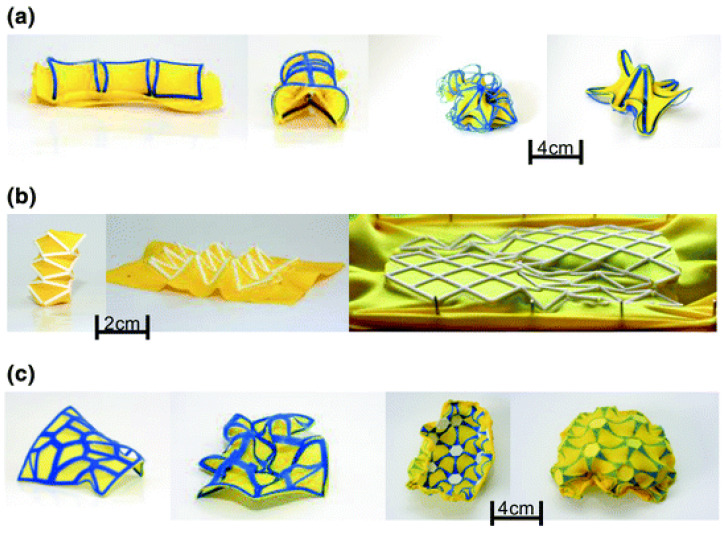
3D-printed textile hybrid structures based on bionics, metamaterials, and architecture concepts [59]: (**a**) based on bionics, (**b**) based on metamaterials, and (**c**) based on architecture. Reproduced from [59], with permission from Springer Nature and Copyright Clearance Center, 2023.

4D printing textile polymers store energy in the textile material before printing and then release that energy (recovery) to affect the form and function of printed prototypes [9]. As described in the previous section, PLA filaments are suitable for 3D printing complex parts that can interact under certain conditions because this biodegradable polymer has shape memory characteristics [91]. In this way, PLA filaments printed on synthetic fabrics can be used to create intelligent hybrid textiles, and the fabric can take on different shapes and return to its original shape depending on certain actions, such as temperature or electricity. Similar experiments have been conducted with conductive, functional fabrics that store or transmit energy to the 3D-printed structure [59]. Figure 6 shows a polyester/lycra jersey fabric (yellow color) and a thermoplastic elastomer (TPU)-printed structure by FDM (blue color, in Figure 6a,c). Figure 6b presents the same polyester fabric with a printed PLA structure (white in Figure 6b). The printed structures followed theoretical patterns based on bionics, metamaterials, and architecture.

**Figure 7:**
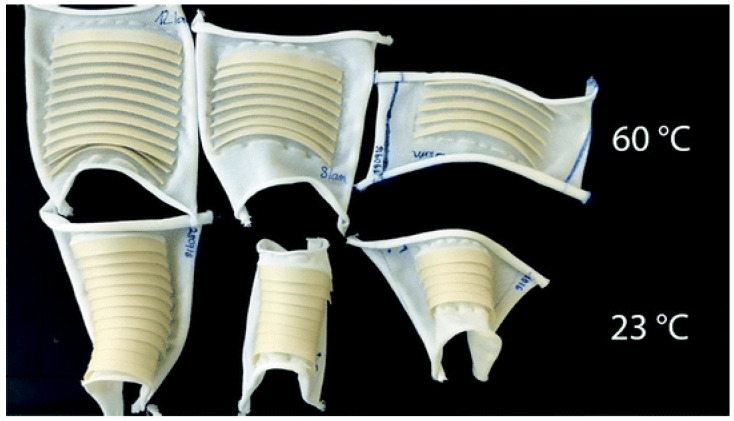
4D textile with and without stimulus: heat [59]. Reproduced from [59], with permission from Springer Nature and Copyright Clearance Center, 2023.

Schmelzeisen et al. [59] developed microstructures printed on previously stretched hyperelastic textiles, which act as energy storage, to measure the change in shape from temperature, as shown in Figure 7. The functional principle of 4D printing polymer textiles is based on the interaction of the properties of an elastically stretched textile surface and shaped beam reinforcements [92]. The textile surface is elastically prestressed and, therefore, absorbs potential energy. Defined areas of the textile are then printed. When the pretension is released, a restorative force is generated in the textile, which is opposed by the elastic stiffness of the reinforcement.

This type of 4D printing polymer textiles has diverse applications since they can act as structural, electronic, piezoresistive, or comfort elements [92,93] and even allow for two-way electronic communication. Thus, 4D printing polymer textiles opens up a new field of application with state-of-the-art technology and multiple material options, since instead of complementing the textile, the textile itself is included in the printed structure [1,94].

4D printing textile polymers can be used in various engineering fields, changing shape depending on the weather or activity. However, to develop multifaceted and multifunctional prototypes, SMPs must be anisotropic, which favors their functionality and adaptation to predefined shape changes under variable conditions. This way, 4D-printed textiles are hybrid, programmable, and active materials [92]. Recent textile 4D printing research examines the interplay between shape memory polymer deposition and soft, stretchy fabric [95]. Among these investigations, Leist et al. [91] used PLA filament deposited on nylon fabrics to thermomechanically train the composites into temporary shapes and restore the original shape through increased temperature. Khan and Hassan [96] showed the 4D textile preparation process with a self-folding box.

Advances in additive manufacturing technology have provided relevant opportunities for manufacturing multidirectional preforms, combining two different polymeric filaments and intertwining them, so that each material provides different properties and acts as programmed under a given action. However, this performance is still in development, but the filament combination bases are mainly based on existing textile configurations. Combining two or more intertwined polymer filaments opens up opportunities for the research and development of 4D-printed multidirectional preforms and composites.

**Figure 8:**
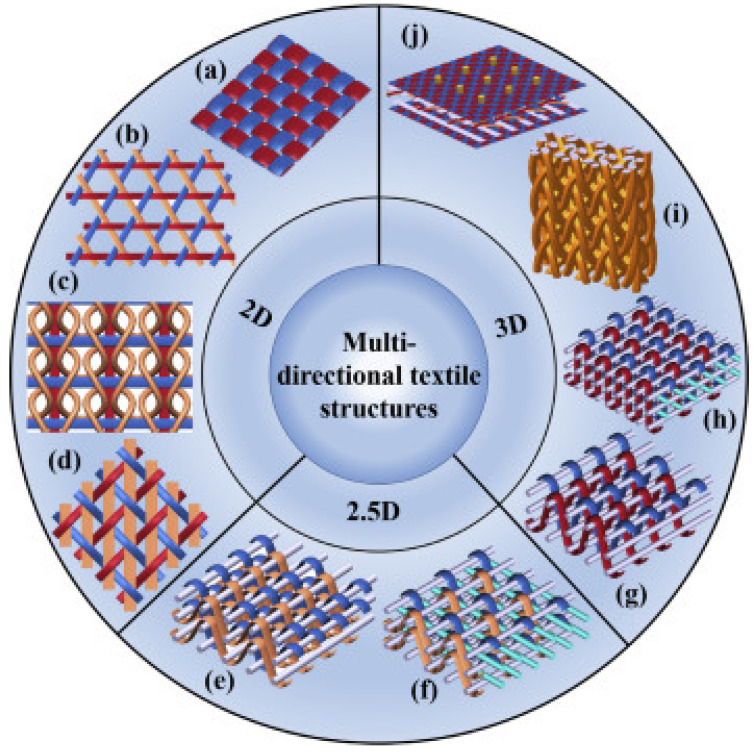
Multidirectional textiles are classified based on the preform structures’ dimension [97]. 2D textile structures: (**a**) plain woven fabric, (**b**) triaxial woven fabric, (**c**) knitting fabric inset with straight yarns, and (**d**) 2D triaxial braid. 2.5D textile structures: layer-to-layer angle-interlock woven fabric (**e**) without, and (**f**) with insetting straight yarns. 3D textile structures: (**g**) 3D through-the- thickness angle-interlock woven fabric, (**h**) 3D orthogonal woven fabric, (**i**) 3D braided structure and (**j**) 3D stitched laminated preform. 2.5D, two-and-a-half- dimensional. Reproduced from [97], with permission from Elsevier and Copyright Clearance Center, 2023.

Multidirectional textile preforms have been manufactured by weaving, knitting, braiding, and stitching techniques with reinforcing yarns oriented in multiple directions [97]. Liu and Chou [97] highlight the classification of typical fabric structures based on multidirectional textile fabrics, which allows for the visualization of the range of fabric options or preforms that can be manufactured based on the spatial orientation of the 3D-printed filaments [98,99], as can be seen in Figure 8. Some of the textile preforms are used to form laminates, the configuration of which is commonly used in fiber-reinforced polymer composites (FRPs). Plain weave is the most widely used and well-known fabric configuration, although triaxial fabrics with inserted or braided stitches are also used. Therefore, the structures can be 2D preforms or 3D-printed configurations with excellent dimensional stability and mechanical performance [100,101]. Two-and-a-half dimensional textile is a family of angled interlock fabrics without full-thickness yarns.

The extensive knowledge gained in 3D printing has allowed for the development of multidirectional preforms with complex geometries using pure ABS filament [102]. The first preforms to be manufactured under FDM technology were topologically independent orthogonal preforms [103].

There are several alternatives to prepare intelligent materials printed in 3D that can modify their geometric shape under external conditions. One is to coat the fabrics with a polymer with shape memory capacity. Polyurethane (PU) is an elastomeric polymer that has shape memory. Liem et al. [84] used PU combined with dimethyloldihydroxyethylene urea (DMDHEU) as a fiber coating on cotton fabrics to reduce residual stress in the fabric. Cotton fabrics are considered natural, absorbing moisture and increasing their volume. In this sense, the shape memory change in these cotton fabrics was attributed to tissue swelling promoted by urea. This technique has been used with other materials, such as polyester [85]. Likewise, the technique has been transferred to other objectives, such as achieving intelligent synthetic fabrics that restrict the presence of wrinkles [86] or other permeable effects depending on the temperature and relative humidity to achieve thermal and moisture-insulating fabrics [104,105].

Using nanoparticles increases the potential of 4D printing to design SMP compounds. Carbon nanotubes enable the development of intelligent fabrics. By using PU reinforced with carbon nanotubes, it is possible to change the shape of the textile preform depending on the temperature when single-walled carbon nanotubes (SWCNTs) are used, as they are highly conductive. Another alternative is to carry out simultaneous 3D printing of preforms using an SMP material and a conductive material, such as silver paste [106].

3D printing technologies have advanced substantially, allowing disruptive technologies to be used in forming 4D-printed structures. In this sense, Wang et al. [59] used a twin-nozzle conversion printer to impregnate a continuous carbon fiber with polyamide (PA 6.6) in the nozzle before printing. This technology obtains a bilayer filament, where the materials act independently since they are not mixed. Thus, one layer or face of the filament is PA 6.6, and the other is carbon fiber. The structural rigidity of the carbon fiber exerts a significant force on the PA 6.6, which causes the polymer to move or rotate according to the temperature induced in the printed preform. This implies that 4D-printed textiles can be made without SMP materials. Some researchers are conducting remarkable work with this type of multilayer filament, developing and deepening the fundamental vision of 4D textiles [59,92].

## 6. Applications

**Figure 9:**
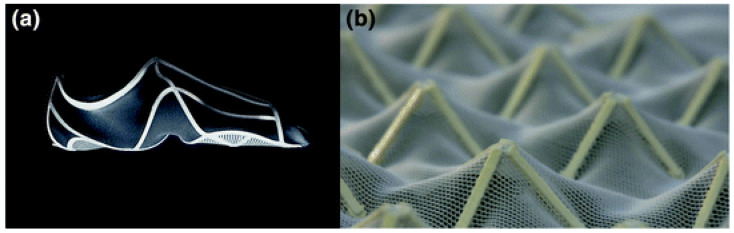
Some 4D printing industrial applications: (**a**) adaptive shoes and (**b**) sound-absorbing panels [59]. Reproduced from [59], with permission from Springer Nature and Copyright Clearance Center, 2023.

This work discusses the advantages of high customization and functional adjustment of structures or preforms manufactured in 4D printing. In this way, the potential of these intelligent materials is very high, and they can be used in various industrial sectors such as aerospace, robotics, instrumentation, automotive, medical products, sports, or clothing, among others. 4D textiles are in the prototyping stage focused on changing dimensions or geometry. The preforms focus on sound absorption applications or custom shoe fit [59,70,92], as shown in Figure 9.

In order to achieve these advances in 4D printing, research has been heavily focused on using SMP materials. In this way, the following advanced applications are found:

### 6.1. Breathable Fabrics

This type of fabric requires the use of temperature-sensitive SMP materials. Similar to what was described in the previous section, these materials are applied to the textile to develop anti-wrinkle or anti-shrinkage fabrics. These properties make them highly desired products for the textile sector. The most widely used material is PU, which, when combined with nanoparticles, acquires the capacity of intelligent material with shape memory (SMPU). The use of SMPU on textiles also allows for the regulation of the transfer of heat and moisture. The water vapor permeability (WVP) of SMPs is regulated by human body temperature. Various authors have studied and evaluated this mechanism [107], finding that the relationship plot of WVP with temperature fits perfectly with the Arrhenius relationship and, therefore, is dramatically influenced by both hydrophilic group content and soft segment crystal melting of PU [108].

### 6.2. Shock Absorbing Fabrics

SMP materials have damping properties related to their glass transition temperature (Tg), which is why they are used in applications requiring them to absorb impact energy. One of the first applications oriented in this direction was the manufacture of fabrics for automobile seatbelts using filaments of block copolymers of poly(ethylene terephthalate)-poly(caprolactone) with shape memory. In this way, SMP fabrics can absorb kinetic energy and increase passenger safety [108,109].

### 6.3. Intelligent Textiles for Energy Storage

**Figure 10:**
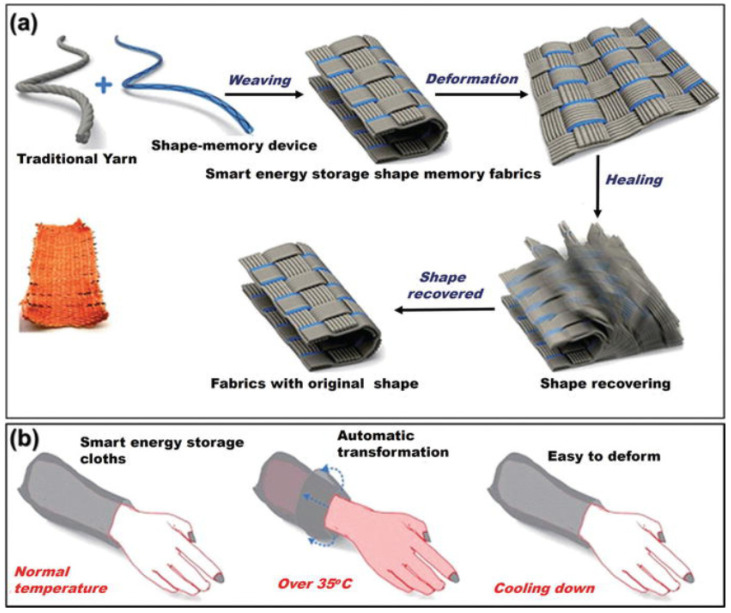
Schematic representation of: (**a**) SC shape memory woven into a conventional fabric and (**b**) shape memory smart textile. Reproduced from [110], with permission from Royal Society of Chemistry, 2023.

The fourth industrial revolution brings with it the use of portable devices, an emerging market that develops devices based on the Internet of Things (IoT). Portable electronic devices use radio technology and include the following products: Smartwatch, fitness bracelets, virtual reality glasses, and health and wellness devices. Electronic circuits are eventually integrated into natural or synthetic textiles to develop smart wearable electronic devices. One of this device’s main objectives is the energy storage capacity, which depends on the electrodes, conductivity, and specific surface area. Huang et al. [110] used an SMP material as a fiber core to fabricate temperature-sensitive electrodes. The shape memory capacity allows two of these fabricated electrodes to be twisted to form a supercapacitor SC (Figure 10).

### 6.4. Origami

Additive manufacturing technology is constantly developing, so it is possible to make significant advances toward 4D printing. In this sense, the art of origami, additive manufacturing, and nanomaterials merge to transform a 3D model into a printed preform that shrinks with heat. The polymer contracts and transforms into a 3D object upon immersion of the printed preform in hot water. This new 4D printing method has the potential to significantly impact various fields of knowledge for developing functional devices. This flexible method allows for the use of functional materials such as conductive or magnetic inks.

**Figure 11:**
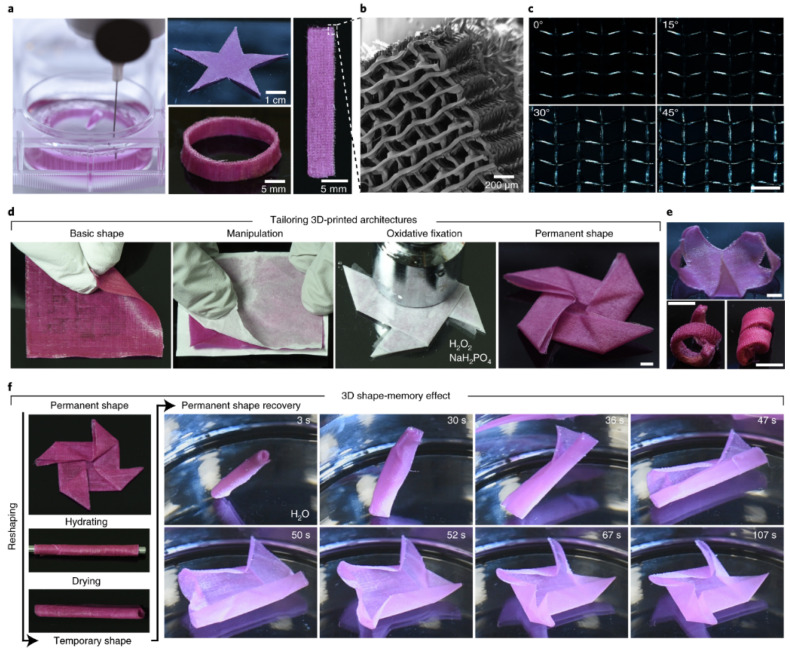
Images of basic 3D-printed keratin architectures [111]: (**a**) Photograph illustrating the 3D printing process that uses the extracted keratin as an ink, (**b**) SEM micrographs of 3D structure of the keratin architecture that is composed of aligned and stacked fibres deposited in a rectilinear pattern, (**c**) polarized light microscopy images of a rectilinear 3D-printed pattern (scale bar, 400 μm), (**d**) sequence of photographs illustrating the post-3D-printing process that is implemented to tailor the shape of basic 3D-printed structures (scale bar, 5 mm), (**e**) photographs of a star with bent arms (**top**) and of spirals (**bottom**) that were obtained by post-3D-printing processing (scale bars, 1 cm), (**f**) photographs showing the WTSM effect in 3D architectures, which is illustrated with a star-shaped origami model. Reproduced from [111], with permission from Springer Nature and Copyright Clearance Center, 2023.

Luca Cera et al. [111] developed a system based on hierarchically structured keratin with shape memory and moisture-sensitive properties. The authors designed and fabricated more complex hierarchical shape–memory structures with a unidirectional shape–memory modality. The scaffolding was built by 3D printing. These 4D printing structures are focused on bioengineering and smart textiles [112], as shown in Figure 11. The frameworks were imprinted using extrusion needles (with minimal diameters), using the thinning properties of keratin. This strategy enabled the production of 3D-printed volumes with highly ordered architectures with an inherent structural hierarchy. The authors designed a star-shaped origami model to illustrate the efficiency of the shape memory mechanism. In this way, when the material is hydrated, the origami becomes malleable and unfolds; when it is dehydrated, it locks into its new geometric shape. This experimentation demonstrates the capacity of 4D printing as a sustainable technology, which allows for the manufacturing of synthetic or biodegradable smart textiles, such as garments, that can adapt to the body or gears that absorb tension energy.

### 6.5. Smart Wearable Electronics

Flexible strain sensors effectively convert mechanical deformation into electrical signals that can detect movements and monitor human–computer interaction in the medical field [113,114,115]. Flexible strain sensors are developed using conductive hydrogels due to their flexibility and conductive ability [116,117,118]. However, the effective development of hydrogel sensors requires suitable 3D printing inks to achieve injectability [119,120].

Shi Feng et al. [121] developed a thermoresponsive composite hydrogel through a simple single-pot freeze–thaw method to construct an interpenetrating network. The sensor has self-healing capabilities and can be 3D-printed. The researchers managed to monitor different human body movements, including large movements (fingers, wrist, and elbow) and small movements (deep breathing).

**Figure 12:**
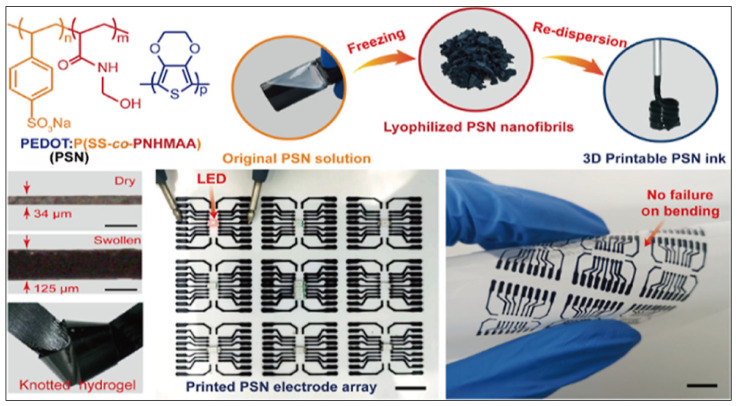
Graphical abstract of novel PEDOT:PSS ink with superior 3D printability for direct ink writing 3D printing [122]. DIW 3D printing of the PSN hydrogel (**upper**). Volume change from dry film to hydrogel and PSN hydrogels upon knotting distortion (**lower left**). Lighting up of an LED with the 3D-printed PSN hydrogel-based electrode arrays (**lower center**). Bending of the 3D-printed PSN electrode arrays without failure/delamination (**lower right**). Scale bars: 5 mm. Reproduced from [122], with permission from American Chemical Society, 2024.

Hydrogels based on poly(3,4-ethylenedioxythiophene):poly(styrene sulfonate) (PEDOT:PSS) are used for bioelectronic applications, although simultaneously achieving high conductivity, robust mechanical properties, and accessible 3D printing is challenging. Jiawen Yu et al. [122] developed highly conductive, intrinsically soft, strong yet stretchable PEDOT:PSS hydrogels through a simple PSS chain engineering strategy to introduce thermally cross-linkable N-(hydroxymethyl)acrylamide segments. The authors found that the resulting PEDOT:PSS hydrogel (Figure 12) exhibits high electrical conductivity, high stretchability (>50%), low Young’s modulus, and high toughness.

## 7. Prospects and Conclusions

Technological advances and the development of new and advanced materials allow the transition from 3D printing to 4D printing innovation. Using materials developed in laboratories demonstrates the potential of 4D printing, which is still only in its infancy. 4D printing makes it possible to manufacture actuators and sensors for various technological applications. Its most significant use is in intelligent textiles, wearable technologies, and auxiliary medical devices.

The potential of 4D printing lies in modular manufacturing, where fabric-printed material interaction enables the creation of bio-inspired and biomimetic devices. The step of 3D printing and its innovative complement in 4D printing entails additive manufacturing as more versatile since it can handle and combine a diversity of materials and additive manufacturing technologies to design and manufacture complex (biomimetic) and independent geometries simply.

The next step is effectively integrating additive manufacturing techniques with other manufacturing techniques to overcome the challenges of build time, dimensional control, and functionality of manufactured parts.

Innovative fabrics are the first step towards this complementary technological transition. The developed prototypes allow for diversification in their commercialization, and the scope of industrial applications is still largely unexplored. SMP materials require more precise control over the range of strain they can achieve, involving numerical simulations, molecular dynamics, and mesoscale mechanics.

In any case, SMP materials offer tremendous current and potential opportunities in the textile industry for developing smart clothing for protection against extreme environments, auxiliary prosthetics, or comfort medical devices. Textiles can play much more significant roles in the future, far beyond what is currently being achieved.

## Data Availability

This study does not report any data.
